# Can miRNAs be useful biomarkers in improving prognostic stratification in endometrial cancer patients? An update review

**DOI:** 10.1002/ijc.33857

**Published:** 2021-11-17

**Authors:** Gloria Ravegnini, Francesca Gorini, Eugenia De Crescenzo, Antonio De Leo, Dario De Biase, Marco Di Stanislao, Patrizia Hrelia, Sabrina Angelini, Pierandrea De Iaco, Anna Myriam Perrone

**Affiliations:** ^1^ Department of Pharmacy and Biotechnology University of Bologna Bologna Italy; ^2^ Division of Oncologic Gynecology IRCCS Azienda Ospedaliero‐Universitaria di Bologna Bologna Italy; ^3^ Department of Medical and Surgical Sciences, DIMEC University of Bologna Bologna Italy; ^4^ Department of Experimental, Diagnostic and Specialty Medicine University of Bologna Bologna Italy; ^5^ Pathology Unit, IRCCS Azienda Ospedaliero‐Universitaria di Bologna Bologna Italy; ^6^ Centro di Studio e Ricerca delle Neoplasie Ginecologiche University of Bologna Bologna Italy

**Keywords:** endometrial cancer, miRNAs, prognostic and diagnostic biomarkers, personalized medicine

## Abstract

Endometrial cancer (EC) is the most common gynecological cancer, with annual incidence rates in Western countries ranging between 15 and 25 per 100 000 women. About 15% to 20% of patients with EC have high‐risk disease and follow an aggressive clinical course. Unfortunately, the assessment of histologic parameters is poorly reproducible and conventional clinicopathological and molecular features do not reliably predict either the patient's response to the available treatments or the definition of personalized therapeutic approaches. In this context, the identification of novel diagnostic and prognostic biomarkers, which can be integrated in the current classification schemes, represents an unmet clinical need and an important challenge. miRNAs are key players in cancer by regulating the expression of specific target genes. Their role in EC, in association with clinical and prognostic tumor biomarkers, has been investigated but, so far, with little consensus among the studies. The present review aims to describe the recent advances in miRNAs research in EC taking into consideration the current classification schemes and to highlight the most promising miRNAs. Finally, a perspective point of view sheds light on the challenges ahead in the landscape of EC.

## Graphical abstract

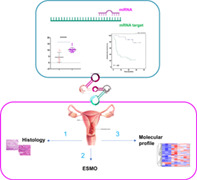

**Abbreviations**
CCC: clear cell carcinoma
CS: carcinosarcomas
EC: endometrial cancer
EEC: endometrioid endometrial cancer
EMT: epithelial‐mesenchymal transition
ESGO: European Society of Gynaecological Oncology
ESMO: European Society of Medical Oncology
FIGO: International Federation of Gynecology and Obstetrics
GOG: Gynecologic Oncology Group
lncRNAs: long noncoding RNAs
miRNA: microRNA
MMRd: mismatch repair deficiency
ncRNAs: noncoding RNAs
NSMP: no specific molecular profile
ProMisE: Proactive Molecular Risk Classifier for Endometrial Cancer
SEC: serous endometrial cancer
TCGA: The Cancer Genome Atlas

## 1 INTRODUCTION

Endometrial cancer (EC) is the most common gynecologic cancer in the developed countries with annual incidence rates of about 15 to 25 per 100 000 women.^1^, ^2^, ^3^ Risk factors are represented by the triad of metabolic syndrome: obesity, diabetes and hypertension; moreover, additional genetics and epigenetics factors can play a major role in EC etiology, associated to hormonal factors such as nulliparity, polycystic ovarian syndrome and all causes of hyper‐estrogenism not balanced by progesterone/progestin presence. This frame describes a multifactorial and heterogeneous disease, in which the prognosis is based on surgical and pathological factors: International Federation of Gynecology and Obstetrics (FIGO) stage, grading and histotype, depth of myometrial invasion, lymphovascular space invasion (LVSI) and lymph node metastases (LNM).

Currently, the stratification risk system appears to be insufficient and inadequately informative in current clinical practice and often it presents challenges in identifying the most appropriate therapeutic approach. Eventually, histopathological parameters used to identify risk factors are not always easily reproducible, particularly in high‐grade carcinomas with intratumoral heterogeneity. All these factors suggest that EC is inappropriately treated due to subjective interpretation of clinicopathological characteristics even by experienced clinicians.^4^, ^5^, ^6^, ^7^, ^8^, ^9^, ^10^, ^11^, ^12^

A new classification based on the molecular characteristics of the EC was proposed in 2013 by The Cancer Genome Atlas (TCGA), however this classification is complex to reproduce in a clinical laboratory and results in a poor clinical translation, despite the surrogate molecular analyses proposed independently by the Vancouver and European Groups.^13^ In this context, the identification of novel biomarkers remains an unmet clinical need and an appealing opportunity is represented by the small non coding RNAs (ncRNAs), in particular, microRNAs (miRNAs). In the last decade, the research on miRNAs in EC have increasingly grown; nevertheless, the integration of miRNAs results with the proposed clinical and molecular classifications is very limited, leaving an important knowledge‐gap.

## 2 CLASSIFICATION SCHEMES IN EC

**FIGURE 1:**
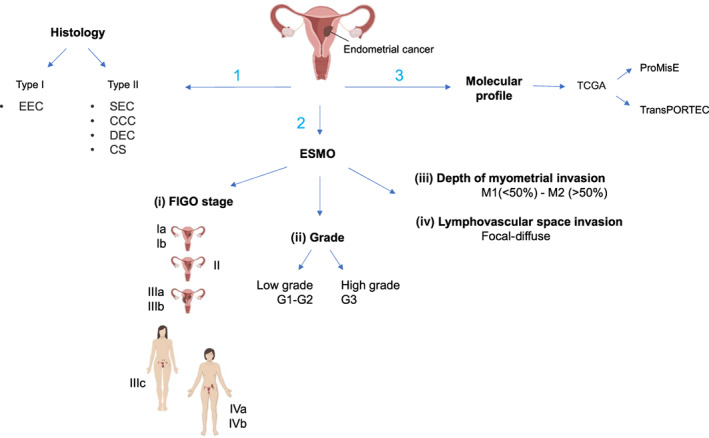
Summary of the current classification schemes in EC. There are three different types of classification: (1) histological (Type I and Type II), (2) histopathologic based on ESMO risk and (3) molecular based on TCGA. Type I includes endometriod EC whereas Type II includes serous carcinomas (SEC), clear cell carcinomas (CCC), dedifferentiated endometrial carcinoma (DEC) and carcinosarcoma (CS). ESMO has identified specific prognostic factors (i, FIGO stage [IA, IB, II, IIIA, IIIB, IVA and IVB]; ii, grade [Grade 1‐3]; iii, depth of myometrial invasion; iv, lymphovascular space invasion) based on which clinicians stratify patients into four distinct risk groups (low, intermediate, intermediate‐high and high risk). From a molecular point of view, TCGA has proposed a new classification that has been then implemented by the ProMisE and TransPORTEC algorithms. EC, endometrial cancer; EEC, endometriod EC; ESMO, European Society of Medical Oncology; FIGO, International Federation of Gynecology and Obstetrics; ProMisE, Proactive Molecular Risk Classifier for Endometrial Cancer; TCGA, The Cancer Genome Atlas [Color figure can be viewed at wileyonlinelibrary.com]

Classification schemes in EC are multiple and complex (Figure 1). Presently, there are three different types of classifications: histological (Type I and Type II), histopathologic based on European Society of Medical Oncology (ESMO) risk and molecular based on TCGA.

### 2.1 Histological classification

For years, this disease has been simply divided and trivialized into two main groups: estrogen‐dependent (Type I) and estrogen‐independent (Type II). Type I, low and intermediate grade endometriod ECs (EECs), is the most common (70%), it is associated with hormone receptor positivity^4^, ^5^ and, generally, it has a favorable prognosis. Type II includes high grade EECs and nonendometrioid subtypes such as serous (SEC), clear cell (CCC), carcinosarcomas (CS) and undifferentiated EC. These tumors are estrogen‐independent, not associated with obesity and with a poor prognosis.^4^ However, it is now clear that this simple dichotomous division is not adequate to represent the complex heterogeneity of this tumor. Generally, EC is considered a cancer with a favorable prognosis, therefore, far from the well‐defined diagnostic‐therapeutic structure of ovarian carcinoma, rarer and more lethal. But over time, it has been realized that not all ECs have a favorable prognosis, and some of them resemble ovarian carcinoma.^6^ This happens not only for Type II but also for some Type I tumors that sometimes show a surprisingly unexpected aggressive behavior.

### 2.2 Classification based on the ESMO risk

The ESMO has identified specific prognostic factors (i, FIGO stage; ii, grade; iii, depth of myometrial invasion; iv, LVSI) based on which clinicians stratify patients into four distinct risk groups (low, intermediate, intermediate‐high and high risk),^7^, ^8^, ^9^ and define treatment. Despite the supposedly differentiated clinical risk factors, there remains a considerable variation in the standardization of therapy. Apparently, few low grade tumors share clinical features with high grade tumors, developing a more aggressive disease with high rates of distant metastasis and poor prognosis, and, on the other hand, some other patients with high risk factors show an impressively long progression‐free survival.^10^ Moreover, histotype and tumor grade are parameters weakly reproducible among pathologists, with significant interobserver variations and this is most pronounced in high‐grade and particularly in heterogeneous tumors.^11^, ^12^

### 2.3 Molecular classification

Due to the heterogeneity of this cancer and the challenge of categorizing the single patient in a predefined risk group, a novel classification of EC based on molecular parameters has been proposed. In 2013, the TCGA endometrial collaborative project recognized four different prognostic groups: (a) the ultramutated subtype that encompassed *POLE* exonuclease domain mutated (POLE) cases, with an excellent prognosis; (b) the hypermutated subtype, characterized by mismatch repair deficiency (MMRd), with an intermediate prognosis; (c) the copy‐number high subtype, with p53 abnormal/mutated features (p53abn), characterized by poor prognosis; (d) the copy‐number low subtype, also known as No Specific Molecular Profile (NSMP), with an intermediate prognosis.^13^ The TCGA highlighted that EC is a group of tumors sharing genomic characteristics with serous ovarian cancer, basal‐like breast cancers and colorectal cancer, rather than a single entity. Furthermore, from a molecular point of view, some endometrioid and serous tumors are distinct, while others are more similar, suggesting that these tumors may have benefits from similar treatments. Conversely, even in this circumstance, the direct clinical translation has proven to be difficult and improvement of the TCGA classification have been suggested. In particular, two research teams proposed and validated the molecular classifiers using surrogate markers. The Proactive Molecular Risk Classifier for Endometrial Cancer (ProMisE) algorithm evaluates *POLE* mutation, p53 and MMR expression analyses to sequentially assess the MMRd group, then *POLE* mutant and finally aberrant p53 cases; the remaining tumors are considered p53 normal.^14^, ^15^ Similarly, the TransPORTEC has recognized four prognostic subsets, diagnosing first *POLE* mutant tumors, then MMRd tumors, *p53*‐mutant tumors and ECs with NSMP.^16^ In both the cases, after ECs stratification based on these molecular features, prognostic signatures emerged. This prompted to a diagnostic algorithm that includes molecular criteria, resulting in a novel, more unbiassed and clinically meaningful EC classification. The clinical and prognostic relevance of these molecular subgroups has been corroborated in several studies^14^, ^17^, ^18^, ^19^ and the most recent ESGO guidelines recommend an integration of standard clinicopathological parameters and new risk profiles, suggesting molecular characterization in all endometrial carcinomas, especially those at high risk.^20^ However, some histopathological parameters, independently by the molecular subtype, as stage and LVSI, do not have a molecular surrogate and are still critical in the pathological evaluation. Ideally, molecular and clinicopathologic prognostic classification schemes work better together. Unfortunately, the proposed molecular system remains difficult to apply to the routine clinical management as it involves laboratory equipments to carry out definite molecular and pathological analyses which, often, are not available in the peripherical facilities. Moreover, recent studies proposed additional classes to be implemented in the recent classification in order to further stratify the risk of relapse.

## 3 MiRNAs

Since the ENCODE project highlighted that about 75% of the genome is transcribed in RNAs and only 3% is represented by gene encoding for proteins, the research has made several advances and the ncRNAs have been recognized as key players in many biological processes.^21^, ^22^ MiRNAs, in particular, are small ncRNAs of about 19 to 24 nucleotides (nts) which play an important role in modulating the expression of their targets at the post‐transcriptional level. This regulation is mediated by base pairing to a 6 ‐ 8 nts sequence of the mRNA target, with perfect or imperfect complementarity and leads to expression inhibition by mRNA degradation or translational repression; a single miRNA can modulate the expression of hundreds of mRNA targets and, conversely, a mRNA target may be regulated by multiple miRNAs.^23^, ^24^, ^25^, ^26^ In the last two decades, the research has progressively characterized miRNAs function and mechanism of action to finely regulate the target genes. Today, it is well recognized that miRNAs play a pivotal role in several processes, such as cell growth development, cell cycle, apoptosis and many others. Compelling evidences have established that miRNA expression is dysregulated in human diseases, including cancer. Of note, miRNAs may act as oncogenes or tumor suppressors and, depending on cellular context, the same miRNA may behave in different ways. Therefore, deregulation of miRNAs can affect the hallmarks of cancer, including sustaining proliferation, evading apoptosis and resisting cell death, promoting invasion and metastasis and inducing angiogenesis.^27^, ^28^

Over the past decade, with the research advances, a plethora of deregulated miRNAs have been progressively described in a wide range of solid and liquid malignancies; this highlights the importance of these epigenetic regulators in fine tuning the biological processes.^24^, ^25^

## 4 MiRNAs IN EC

In recent years, many studies have identified different genes and miRNAs as potential biomarkers in EC.^29^, ^30^, ^31^, ^32^, ^33^, ^34^, ^35^ However, in most of these, especially when focused on miRNAs, deregulation was observed by comparing tumor with normal counterpart or healthy tissue.^36^ In this context, among others, a complete overview is presented by Srivastava et al, who reviewed the miRNAs differentially expressed in EC with respect to normal endometrial tissue.^37^ On the contrary, the studies that analyzed miRNA expression taking into account other parameters, such as grade or the existing classifications schemes, particularly the TCGA one, are limited and with small consensus.

Given these premises, the aim of this narrative review is to provide an overview of the miRNAs that have been identified as significantly associated with EC molecular and clinical features and that could be potentially integrated in the available classifications.

To this purpose we searched for papers analyzing miRNA expression in EC in relation to prognostic and molecular classifications; reports comparing miRNAs between EC and normal tissue were not considered because out of our scope.

The papers included in this revision are summarized in Table 1, whereas Table 2 reports the main role and potential targets of miRNAs described in the above‐mentioned papers. In our review, first, we focused on studies that analyzed tissue miRNAs and prognostic factors and, subsequently, on the ones investigating the potential association between circulating miRNAs and prognostic factors, based on the current classifications.

**TABLE 1:** Summary of the studies included in the present review

Authors, year, reference	Aim of the study	Number of cases (n) and groups	miRNA	Main results	*P*‐Value
MiRNAs associated with stage and grade					
Chung et al, 2009^46^	To identify miRNAs associated with EEC	30 ECs n = 25 Stage I‐II, n = 5 Stage III	miR‐200a, miR‐205	↑ miR‐205 and miR‐200a in advanced stage ECs	<.05
Torres et al, 2013^47^	To define diagnostic and prognostic miRNAs in EEC	77 ECs n = 32 Stage IA, n = 18 IB, n = 5 II, n = 2 IIIA, n = 3 IIIB, n = 10 IIIC1, n = 5 IIIC2, n = 1 IVA, n = 1 IVB	miR‐200a, miR‐200b, miR‐429	↑ miR‐200a, miR‐200b, miR‐429 in earlier stage	<.05
Tsukamoto et al, 2014^49^	To identify a set of miRNAs associated with clinicopathological characteristics	28 ECCs n = 15 Grade 1, n = 11 Grade 2, n = 2 Grade 3; n = 9 Stage IA, n = 5 Stage IB, n = 1 Stage II, n = 4 Stage IIIA, n = 9 Stage IIIC	miR‐205	↑ level in Grade 2 and 3 vs Grade 1	.024
			miR‐499	↓ level in Stage IA‐IB vs more advanced	.02
				↓ level in Stage IA and Grade 1 vs others (Stage IB or more advanced and Grade 2 or 3)	<.05
Wilczynski et al, 2016^48^	To evaluate miR‐205 expression in regard to patients' clinical and histopathological features	90 ECs n = 62 Stage I‐II, n = 28 III‐IV n = 34 Grade 1, n = 42 Grade 2, n = 14 Grade 3	miR‐205	↑ in early stage EC pts compared to advanced stage ↓ in poorly differentiated (G3) tumors compared to moderately differentiated	.045 .02
Canlorbe et al, 2016^60^	To evaluate if miRNA profiles of Grade 1‐2 ECs are related to nodal status and can be used as a tool to adapt surgical staging	36 ECs (Grade 1‐2): n = 9 LN+, n = 27 LN−	miR‐184, miR‐34b‐5p, miR‐34c‐5p, miR‐34c‐3p, miR‐375	↓ in ECs with LN+ vs ECs with LN‐	<.05
Yang et al, 2018^50^	To investigate role of miR‐210 in EC	66 ECs n = 49 Stage I, n = 7 Stage II, n = 10 Stage III	miR‐210	↓ level in Stage I than in Stage II‐II	<.001
Wilczynski et al, 2018^45^	To verify clinical usefulness of miR‐200c in EEC	90 EECs n = 49: Stage I, n = 13 Stage II, n = 21 Stage III, n = 7 Stage IV	miR‐200c	↑ in early stage (I‐II) compared to advance stage (III‐IV)	.01
Hu et al, 2019^54^	To investigate miR‐449a in EC	40 ECs n = 28 Stage I‐II, n = 12 Stage III‐IV	miR‐449a	↓ level in Stage III‐IV stage vs in I‐II	<.05
Kalinkova et al, 2020^58^	To characterize miRNA expression in Grades 1 and 3 EEC and SEC	62 ECs n = 41 EECs, n = 21 SEC EECs → n = 20 Grade 1, n = 21 Grade 3	let‐7c‐5p	↓ in Grade 3 EECs vs Grade I EECs	.003
			miR‐125b‐5p	↓ in Grade 3 EECs vs Grade I EECs	.012
			miR‐23b‐3p	↓ in Grade 3 EECs vs Grade I EECs	.002
			miR‐99a‐5p	↓ in Grade 3 EECs vs Grade I EECs	.011
			let‐7 g‐5p	↓ in SEC vs EEC	.005
			miR‐195‐5p	↓ in SEC vs EEC	.022
			miR‐34a‐5p	↓ in SEC vs EEC	.001
			miR‐497‐5p	↓ in SEC vs EEC	<.0001
Fridrichova et al, 2020^59^	To investigate the relevance of miRNA profiles in EC stratification	182 ECs (of which 62 from reference 58) n = 92 EEC, n = 44 SEC, n = 21 CS, n = 20 CCC	miR‐497‐5p	↑ miR‐497‐5p in EECs vs others ↓ miR‐497‐5p in high vs low grade ↓ miR‐497‐5p in advanced ECs (IIIA+IIIB+IIIC1 + IIIC2 + IVB) vs IA + IB + II ↓ miR‐497‐5p in ECs with LN and distant metastases (IIIC1 + IIIC2 + IVB) vs IA + IB + II + IIIA+IIIB	<.001 <.001 <.001
Wang et al, 2020^64^	To explore diagnostic and prognostic miRNA markers in EC	• 387 ECs (TCGA‐UCEC) n = 258 training dataset; n = 129 validation dataset • 17 ECs from GSE35794	Model of 5 miRNAs (miR‐128‐3p, miR‐106a‐5p, miR‐7706, miR‐18b‐3p, miR‐455‐5p)	The model was more effective in stratifying EC patients at high risk compared to the FIGO stage Significant association between the prognostic model and the TCGA molecular scheme	
Fu et al, 2021^61^	To construct a miRNA signature able to predict LNM	Two pts cohorts: (1) 324 ECs (TCGA‐UCEC): n = 226 training set, n = 98 validation set (2) 24 ECs n = 12 LNM +, n = 12 LNM ‐	miR‐34b‐5p, miR‐34c‐3p, miR‐34c‐5p,	↓ in LNM+ vs LNM‐	<.05
Ravegnini et al, 2021^65^	To characterize miRNAs expression in order to better stratify the TCGA intermediate risk ECs	72 ECs (of which n = 15 CTNNB1 mutant): n = 41 NSMP ECs n = 31 MMRd ECs	miR‐499a‐5p	↑ in CTNNB1 mutant ECs	<.0001
		Validation in n = 111 ECs (TCGA cohort, of which n = 30 CTNNB1 mutant): n = 72 NSMP ECs n = 39 MMRd ECs		↑ in CTNNB1 mutant ECs; ECs with high miR‐499a‐5p and wild‐type CTNNB1 have higher risk of death	.0001
					.006
MiRNAs associated with recurrence					
Devor et al, 2017^69^	To identify early recurrence in the course of therapy	54 ECs • n = 18 EECs, n = 18 SEC, n = 18 CS • n = 27 recurrent pts, n = 27 non‐recurrent pts	miR‐181c	↓ in EEC recurrence	.01
		Validation in n = 215 EECs (TCGA cohort): n = 25 recurrent pts, n = 190 non‐recurrent pts			.001
de Foucher et al, 2018^70^	To evaluate whether miRNAs can be correlated with recurrences	21 ESMO low grade ECs n = 7 recurrent pts, n = 14 nonrecurrent pts	miR‐184,	↓ level in recurrent ECs	<.001
			miR‐196b‐3p	↓ level in recurrent ECs	<.05
			miR‐497‐5p,	↓ level in recurrent ECs	<.05
Salinas et al, 2019^72^	To create a prediction model to classify EEC pts into low or high risk using a combination of molecular and clinical‐pathological variables.	127 EECs n = 70 low risk, n = 56 high risk		Clinical parameters alone were less effective in stratifying the pts vs a model combining clinical data with miRNA expression	Model performance: 88% vs 97%
Wang et al, 2020^71^	To identify a new multi‐RNA‐type‐based molecular biomarkers for predicting the RR and RFS	463 ECs (TCGA‐UCEC) n = 232 training dataset, n = 231 validation dataset n = 75 recurrent pts, n = 388 nonrecurrent pts	miR‐184	↑ level in nonrecurrent ECs	<.001
			miR‐4461	↑ level in nonrecurrent ECs	<.001
			miR‐6511b	↓ level in nonrecurrent ECs	<.001
Circulating miRNAs					
Torres et al, 2013^47^	To identify plasma miRNAs associated with clinicopathological characteristics	34 EECs n = 16 Grade 1, n = 18 Grade 2/3 n = 17 Stage IA, n = 17 stage > IA	miR‐9	↓ miR‐9 in Grade I pts vs Grade II + III	<.05
			miR‐449a	↑ miR‐449a in EEC with stage > IA	<.05
Tsukamoto et al, 2014^49^	To identify a set of EEC‐associated plasmatic miRNAs and evaluate their clinical significance	12 ECCs n = 4 Stage IA + Grade 1, n = 8 advanced tumors	miR‐21	↑ in Stage IA and Grade 1	.017
Ghazala et al, 2021^79^	To assess serum expression of miR‐27a and miR‐150‐5p in EC pts	36 ECs n = 9 premenopause; n = 25 postmenopause n = 28 Type I, n = 8 Type II n = 9 Grade 1, n = 22 Grade 2, n = 5 Grade 3 n = 20 Stage I, n = 10 Stage II, n = 4 Stage IIIc	miR‐27a	↑ in Type I ECs vs Type II ECs	<.001
			miR‐150‐5p	↑ in post vs premenopausal	<.001

Abbreviations: ↓, underregulation; ↑, overexpression; +, positive; −, negative; CCC, clear cell carcinoma; CS, carcinosarcoma; EC, endometrial cancer; EEC, endometriod EC; ESMO, European Society for Medical Oncology; LN, lymph node; LNM, lymph node metastases; pt, patient; RR, recurrence risk; RFS, recurrence free survival; SEC, serous EC; UCEC, Uterine Corpus Endometrial Carcinoma; TCGA, The Cancer Genome Atlas.

**TABLE 2:** Potential role or targets of miRNAs proposed by the analyzed papers

miRNA ID	Reference describing the miRNA	Potential role or targets of miRNAs
let‐7c‐5p	Kalinkova et al, 2020^58^	NRAS, PIK3R5, TP53, AKT2, CCND1, APC2, PIK3CA
let‐7g‐5p	Kalinkova et al, 2020^58^	BRAF, NRAS, KRAS, MLH1, TP53, CCND1, CTNNA1, MYC, MAPK1, PIK3R5, AKT2, APC2, PIK3CA
miR‐125b‐5p	Kalinkova et al, 2020^58^	ERBB2, RAF1, AXIN1, TP53, CTNNB1, CTNNA1, AKT1, PIK3CB, PIK3R5, PIK3CD, TCF7
miR‐150‐5p	Ghazala et al, 2021^79^	MUC4, TP53, C‐Myb, ZEB‐1, EGR2, BAK1, SRCIN1, FOXO4, p27, CCDN1, PDCD4, AKT, MMP9
miR‐181c	Devor et al, 2017^69^	NOTCH2
miR‐184	Canlorbe et al, 2016^60^	TNFAIP2, SND1, CDC25A, c‐MYC
	de Foucher et al, 2018^70^	TNFAIP2, SND1, CDC25A, c‐MYC, BCL‐2, AKT/mTORC1 pathway
	Wang et al, 2020^71^	—
miR‐195‐5p	Kalinkova et al, 2020^58^	CDH1, CCND1, CTNNB1, MYC, PIK3CA, GRB2, GSK3B, SOS2, PIK3R‐1/5, RAF1, EGFR, KRAS, AXIN2, SOS1, AKT3, MAP2K1, BRAF
miR‐196‐5p	de Foucher et al, 2018^70^	HOXB7
miR‐200a	Chung et al, 2009^46^	SLC18A2, OLFM1, ATP8A2, TRO, C2orf32, TCF8, FOXC1, FOXA1
	Torres et al, 2012^47^	Role in EMT (by modulation of ZEB‐1/2 and E‐cadherin)
miR‐200b	Torres et al, 2012^47^	Role in EMT (by modulation of ZEB‐1/2 and E‐cadherin)
miR‐200c	Wilczynski et al, 2018^45^	Role in EMT (by modulation of ZEB‐1/2 and E‐cadherin) MALAT1, KDR, BRD7
miR‐205	Chung et al, 2009^46^	Promotion of a more aggressive phenotype PEG3, P2RY14, JPH4, ECM2, S100A2, ZEB‐1/2
	Tsukamoto et al, 2014^49^	PH4, ESRRG, PTEN
	Wilczynski et al, 2016^48^	Role in EMT by targeting PKCε and/or ZEB‐2 PTEN
miR‐21	Tsukamoto et al, 2014^49^	PTEN
miR‐210	Yang et al, 2018^50^	Role in migration/invasion NIFX
miR‐23b‐3p	Kalinkova et al, 2020^58^	RAF1, EGFR, AKT2, CCND1, CTNNB1, MYC, SOS1, FOXO3, PDPK1, PTEN, MAPK1, GSK3B, PIK3R3, BRAF, PIK3CB
miR‐27a	Ghazala et al, 2021^79^	BAX, FOXO1, MAP2K4, AGGF
miR‐34a‐5p	Kalinkova et al, 2020^58^	Role in EMT (by targeting SNAILs) BRAF, MAP2K‐1/2, PIK3R2, TCF7L1, RAF1, EGFR, ARAF, TP53, AKT2, CDH1, CCND1, CTNNB1, AXIN2, MYC, MAPK‐1/3, CASP9, PIK3CA, LEF1, PTEN, GRB2, BA, ELK1, LEF1, ERBB2, PIK3CB, TP53, LCAM1, NOTCH1, DLL1
miR‐34b‐5p, miR‐34c‐3p, miR‐34c‐5p	Canlorbe et al, 2016^60^	Role in EMT (SNAIL‐1/2, basic helix–loop–helix, E47, E2‐2, TWIST‐1/2, ZEB‐1/2), TP53
	Fu et, 2021^61^	Role in EMT (by targeting SNAILs) Role in proliferation, migration and invasion, cell cycle arrest, apoptosis (by targeting E2F3)
miR‐375	Canlorbe et al, 2016^60^	PDK1, JAK2, IGF1R, AEG‐1, PI3K/Akt pathway
miR‐429	Torres et al, 2012^47^	Role in EMT (by modulation of ZEB‐1/2 and E‐cadherin)
miR‐4461	Wang et al, 2020^71^	—
miR‐449a	Hu et al, 2019^50^	Role in migration/invasion SRC, AKT, ERK‐1/2
	Torres et al, 2013^47^	—
miR‐497‐5p	de Foucher et al, 2018^70^	Role in EMT PBX‐2/3, PBX3, YAP, VEGFA, BDNF
	Kalinkova et al, 2020^58^	SOS2, PIK3R‐1/2, AKT2, CCND1, MYC, AKT3, PIK3CA, MAP2K1, MAPK1, GRB2, GSK3B, PIK3R5, RAF1, EGFR, KRAS, CDH1, AXIN2, SOS1, BRAF
	Fridrichova et al, 2020^59^	Role in EMT (by modulation of ZEB‐1/2) MAPK, RAF1, KDR, IGF1‐R, IRS1, CBX4, PDL1
miR‐499	Tsukamoto et al, 2014^49^	—
	Ravegnini et al, 2021^65^	APC
miR‐6511b	Wang et al, 2020^71^	—
miR‐9	Torres et al, 2013^47^	—
miR‐99a‐5p	Kalinkova et al, 2020^58^	CCND1

### 4.1 MiRNAs associated with stage and grade in EC

According to the FIGO^38^ staging system, ECs are divided in four stages based on the tumor spread to other tissue or organs (Stage I to Stage IV) or in three classes based on the on the degree of glandular differentiation (grade).

So far, most of the works have been focused on specific miRNAs, previously identified in other cancer types as key players, with the intent to investigate them in EC.

An interesting miRNA family that seems to be correlated with EC metastatic potential is the miRNA‐200 family.^39^ This family, one of the best characterized, consists of five miRNAs (miR‐200a, miR‐200b, miR‐200c, miR‐141 and miR‐429) which may negatively regulate expression of ZEB‐1/2, that play a critical role in epithelial to mesenchymal transition (EMT).^40^, ^41^, ^42^ In EC, this biological process is particularly important because myometrial invasion is one of the most important prognostic factors for the risk of disease spread outside the uterus, especially LNM. Several studies have reported miR‐200c deregulation in EC tissues compared to the normal counterpart^43^, ^44^; however, its prognostic and clinical significance is more difficult to evaluate. In this regard, Wilczynski et al showed a correlation of miR‐200c with the tumor stage; specifically, the authors analyzed 90 ECs in different stages (49 in Stage I, 13 in Stage II, 21 in Stage III and 7 in IV), and identified an higher expression of miR‐200c in early stage ECs (Stage I‐II) with respect to advanced stages (III‐IV); no additional associations with other prognostic parameters were detected.^45^ Chung and colleagues performed a miRNA profiling in 30 ECs samples and showed that the aberrant expression of miR‐200a was correlated with advanced stage.^46^ The involvement of miR‐200 family in EC was also reported by Torres et al, who investigated it through microarray and real‐time PCR (RT‐PCR) in 77 ECs. In particular, they analyzed miRNA expression in relation to the FIGO stage, showing significant downregulation of miR‐200a and miR‐429 in higher stages.^47^ In addition, they identified a significant association between expression of miR‐200a, miR‐200b, miR‐429 and histological grade. These studies reinforce the idea that miR‐200 family may have a critical role in EMT, which is known to be important in EC metastatic process.^41^ Besides this, other miRNAs are critical players in this process, including miR‐205 which seems to be one of the key‐regulators of EC carcinogenesis and tumor promotion. To clarify this aspect, Wilczynski and colleagues investigated its expression level in 90 ECs. The patients were divided according to FIGO stage (I‐II: n = 62, III‐IV: n = 28) and grade (1: n = 34, 2: n = 42, 3: n = 14). The results showed that miR‐205 was significantly higher expressed in early stage EC patients with respect to advanced stage tumors. In addition, poorly differentiated (Grade 3) tumors presented lower expression compared to moderately differentiated.^48^ Interestingly, the independent study by Chung et al, previously mentioned, showed that aberrant expression of miR‐205, beside miR‐200a, was significantly correlated with advanced stages.^46^ On the contrary, contrasting findings were reported by Tsukamoto and collaborators, who showed that miR‐205 level was higher in Grade 2 and 3 tumors compared to Grade 1 ECs.^49^ Another miRNA potentially involved in EMT and recently explored in EC is miR‐210.^50^ This has shown abnormal expression in several cancers,^51^, ^52^, ^53^ however its function in EC was not clarified. Yang and colleagues analyzed a total of 66 ECs, of which 49 in FIGO Stage I, 7 in Stage II and 10 in Stage III; the same cohort was also divided according the grade and 28 ECs resulted in Grade 1, 28 in Grade 2 and 10 in Grade 3. The expression of miR‐210 was significantly upregulated with the increase of stage and grade; specifically, EC with higher grade and stage showed higher expression of miR‐210. In addition, patients with LNM presented higher expression as well.^50^ Eventually, other miRNAs have been related with EC features, although, these, apparently, have a protective effect. Hu et al recently analyzed the expression of miR‐449a in 40 EC samples, of which 28 in Stage I‐II and 12 in Stage III‐IV.^54^ The selection of this miRNA was due to its potential suppressive effects on cancer initiation and progression.^55^, ^56^, ^57^ miR‐449a expression was analyzed by immunohistochemistry (IHC) and the authors observed a significant lower expression in higher stage ECs compared to Stage I‐II ECs; on the contrary, no difference was detected based on histology or age. Further functional studies demonstrated that miR‐449a could restrain the migration and invasion of EC cells, thus confirming the potential suppressive role of miR‐449a in EC. Kalinkova et al analyzed the expression of a panel of 84 miRNAs in 62 ECs (endometrioid EC: n = 41, of which n = 20: Grade 1 and n = 21: Grade 3; serous EC: n = 21) and 20 normal endometrial specimens.^58^ Comparing Grade 1 and 3 endometrioid EC patients, expression of let‐7c‐5p, miR‐125b‐5p, miR‐23b‐3p and miR‐99a‐5p resulted lower in Grade 3 endometrioid ECs. Moreover, let‐7g‐5p, miR‐195‐5p, miR‐34a‐5p and miR‐497‐5p were significantly downregulated in serous EC with respect to endometrioid EC. In the same year, Fridrichova and colleagues deepened the role of miR‐497‐5p to investigate its relevance in EC patients stratification.^59^ The study cohort of 182 ECs, in part, overlaps the ones presented by Kalinkova et al; specifically, 120 patients were new and 62 already described.^58^ The results displayed a protective role of miR‐497‐5p, highlighting its downregulation in high‐grade tumors, in advanced ECs with metastases and positive for lymph nodes and distant metastases.^59^ Association between LNM and miRNA expression was also investigated by Canlorbe et al.^60^ In particular, the authors evaluated 36 early stage ECs (Grade 1‐2), of which 9 positive and 27 negative for lymph node status. A global miRNA profiling by microarrays showed 12 miRNAs differentially expressed between the two groups; RT‐PCR was used to validate the results in the same cohort and five miRNAs (miR‐34c‐5p, miR‐375, miR‐184, miR‐34c‐3p, miR‐34b‐5p) resulted significantly lower in the Grade 1‐2 EC samples with positive lymph node status compared to those with negative status.^60^ These results are intriguing as ascribe a role to the miR‐34 family in the EC landscape. Recently, its involvement has been also supported by additional evidences^61^; Fu et al analyzed the TCGA—UCEC (Uterine Corpus Endometrial Carcinoma) database with the aim to construct a miRNA signature able to predict LNM. The authors retrieved the data of 324 patients and identified 113 miRNAs differently expressed between the two sets of patients (ie, with and without LNM). The miRNAs potentially associated with LNM were screened by three methods including differentially expressed miRNAs, weighted gene coexpression network analysis and decision tree algorithms. Based on that, 31 miRNAs were tested in the training cohort (n = 226, randomly selected from the TCGA cohort) and, finally, a signature of 15 miRNAs was identified and validated in both training and validation (n = 98) cohorts and risk score for LNM was assed. Eventually, the authors validated their findings in an additional, independent database; among the 15 miRNAs, miR‐34c‐3p, miR‐34c‐5p and miR‐34b‐5p were significantly lower in LNM‐positive ECs compared to LNM‐negative patients. MiR‐34 family deregulation has been widely described in many cancer types, including lung, colorectal, prostate and breast cancer.^62^ Its importance is well recognized along with the development of MRX34, the first cancer therapy based on liposomal miR‐34a mimics (phase I clinical trial, NCT01829971).^63^ MiR‐34 acts as a major player in tumor inhibition through negative regulation of several EMT transcription factors (including SNAIL‐1‐3, ZEB‐1/2, TWIST‐1/2), p53 and additional signal pathways, such as WNT and NOTCH pathways.^62^

In recent years, the approaches based on the available TCGA databases are gaining the research interest and the number of reports is rapidly growing. Beside the work by Fu et al, another example is presented by Wang et al,^64^ who explored the TCGA miRNAs and mRNAs databases. The cohort of 419 samples, of which 32 were normal endometrium, was randomly divided in training and validation set. Initially, they built a diagnostic miRNA classifier to discriminate between EC and normal endometrium tissues; then a prognostic miRNA model for survival prediction in EC patients was generated. Five prognostic miRNAs (miR‐18b‐3p, miR‐106a‐5p, miR‐128‐3p, miR‐455‐5p and miR‐7706) were selected by using a machine learning based approach and Cox regression. The miRNA signature was tested in both training and validation cohorts and two groups of patients were identified; the overall survival (OS) for patients belonging to these two risk groups was estimated by the Kaplan‐Meier curve, showing a significant difference. Interestingly, to evaluate the power of the model, stratification analysis was conducted based on the FIGO stage. The results showed that the miRNA model was able to divide early (Stage I‐II) and advanced stage (Stage III‐IV) EC patients into low‐ and high‐risk groups, respectively, and performed better than the FIGO stage. Of note, the authors also compared the ability of the five miRNAs‐based‐signature in stratifying the four TCGA molecular groups and found a significant association between the prognostic model and the molecular classification.^64^ This aspect reflects the prognostic ability of this model and paves the way for a further improvement of the current TCGA classification scheme. In this context, a recent work by Ravegnini et al, identified the association of miR‐499a the TCGA molecular classes. In particular, miR‐499a‐5p resulted upregulated in the NSMP EC patients harboring *CTNNB1* mutations; the correlation was also corroborated in an independent, larger EC cohort extrapolated by the TCGA cohort. Furthermore, by combining the miRNA expression with the *CTNNB1* mutational status, the authors identified a subgroup of NSMP patients with better OS, and miR‐499a‐5p resulted an independent risk factor of death.^65^

### 4.2 MiRNAs associated with tumor recurrence

As previously mentioned, Type I EEC is the most common histologic subtype. Usually the majority of endometrioid EC patients are considered to be at low risk, when diagnosed at an early stage, with no benefits from further treatment after surgery.^66^, ^67^, ^68^ Though, a subset of these patients shows a higher risk of recurrence and poor overall prognosis. Unfortunately, to date, no accurate tools to identify these high‐risk patients are available and their management remains challenging. Given that, identification of novel biomarkers able to clinically drive the correct patients' selection remains an unmet clinical need. For this reason, researchers have looked with interest to the miRNAs. In this regard, Devor and coworkers analyzed 54 recurrent and nonrecurrent ECs cases from the Gynecologic Oncology Group (GOG) Study‐210.^69^ The study cohort was well balanced, composed of 18 cases for each histological subtype (ie, EEC, SEC an and CS), of which nine were nonrecurrent and nine were recurrent. A miRNA profiling, followed by RT‐PCR validation, identified miR‐181c as significantly downregulated in EEC recurrent cases. Moreover, an independent validation in the TCGA database (n = 25 recurrent vs n = 190 nonrecurrent EECs) confirmed a significant underexpression of miR‐181c among recurrent EECs. An additional value of this work is the functional study in cell models. The authors, indeed, evaluated potential targets of miR181c, first in silico, then on a patients cohort, and eventually in vitro, identifying NOTCH2 as a direct target.^69^

De Foucher et al aimed to explore miRNAs ability to predict recurrence in early stage ECs.^70^ This is a hot topic since ESMO risk classification does not seem sufficiently accurate in predicting risk of recurrence in women with early‐stage EC, and additional biomarkers are strongly wanted. The authors evaluated 21 cases by miRNA microarray analysis, of which 7 tumor sample were from recurrent patients and 14 from nonrecurrent cases. This profiling displayed the significant deregulation of six miRNAs (miR‐184, miR‐195‐5p, miR‐196b‐3p miR‐497‐5p, miR‐6080, miR‐7162‐3p) that were further validated by single assay, but in the same cases. Three miRNAs (miR‐184, miR‐196b‐3p, miR‐497‐5p) showing lower expression in recurrent EC were confirmed.^70^ The results, even if from a small size study cohort, have been in part corroborated by a recent study. Indeed, Wang et al tried to integrate lncRNAs, miRNAs and mRNAs in recurrent and nonrecurrent EC patients in order to identify a multi‐RNA‐type‐based risk score model for predicting the risk of recurrence. To this purpose, the authors analyzed the TCGA data from 463 patients, of which 75 recurrent and 388 nonrecurrent (n = 232 training dataset; n = 231 validation dataset). The generated model was a 13 gene‐based signature (3 miRNAs, 3 lncRNAs, 7 genes) effective for the discrimination of the 5‐year relapse‐free survival (RFS) in the high‐risk and low‐risk patients using the training, validation, and the pooled datasets.^71^ The three miRNAs were miR‐184, miR‐4461 (higher level in nonrecurrent ECs) and miR‐6511b (lower level in nonrecurrent ECs); of these, only miR‐184 was previously described as associated in EC. Overall, the study demonstrated that combination of multiple variables is often more effective than the single parameters by themselves.^71^ The idea of integrating miRNAs was not new and other authors have proposed combined predictive models to assess the risk. In this regard, Salinas et al analyzed 127 EECs, of which 70 defined at low risk and 56 at high risk,^72^ according the criteria and results from GOG 33 and GOG 99 clinical trials.^73^, ^74^ In our study, the authors aimed at developing a prediction model able to have an immediate impact on treatment decisions. Indeed, the authors demonstrated that the clinical parameters alone were less effective in stratifying the patients with respect to a combined model of clinical data with miRNA expression (performance of the models 88% vs 97%, respectively), resulting in potentially useful clinical tests. Even in this case, the work highlighted the importance of integrating multiple clinical and molecular parameters and biomarkers in order to achieve better results in terms of diagnostic or prognostic purpose.

### 4.3 Circulating miRNAs in EC

In the last years, we have witnessed significant advances in the so‐called liquid biopsy from a diagnostic and prognostic point of view. On the same wave, circulating miRNAs have been deeply characterized in many cancer types and EC has not been left out. However, with the aim to outline noninvasive approaches for diagnosis, the majority of the studies focused on EC have, so far, assessed the miRNA levels in plasma or serum samples between oncological patients and healthy individuals^75^, ^76^, ^77^, ^78^ (see Table S1 for a summary). On the contrary, the clinicopathological features, including molecular parameters, staging, grading, and all the other aspects described in this review, have not been widely considered. To date, three studies have considered this specific association. A report by Torres and collaborators, starting from an analysis in EC tissue samples, analyzed 16 plasmatic miRNAs in 34 EEC patients. Among those, miR‐9 was lower in blood samples from Grade 1 patients compared to Grades 2 and 3 and miR‐449a was expressed at higher levels in patients with advanced stages (FIGO stage >IA).^47^ Another work, by Tsukamoto et al, after miRNA expression assessment in EC tissue samples, evaluated miRNA levels in plasma comparing patients stratified according to the FIGO stage and histopathological grade. Plasmatic miR‐21 in four FIGO stage IA and Grade 1 tumors was significantly higher compared to the expression in 8 advanced EC patients. On the contrary, no significant differences in the plasma level of other EEC‐associated miRNAs (miR‐135b, miR‐205 and miR‐30a‐3p) before and after hysterectomy were observed.^49^ More recently, Ghazala and colleagues explored circulating miR‐27a and miR‐150‐5p as potential noninvasive biomarkers in EC. In a secondary analysis the authors estimated the association of these two miRNAs with the clinicopathological features. MiR‐27a showed a significant correlation with the tumor type since it was overexpressed in Type I ECs compared to Type II ECs. MiR‐150‐5p was associated with menopausal state showing overexpression in postmenopausal with regard to premenopausal EC women.^79^ Unfortunately, no studies investigating association between circulating miRNAs and pharmacological response have been currently reported.

## 5 CONCLUSION AND FUTURE PERSPECTIVE

Currently, cancer biopsy represents the gold standard for a precise diagnosis and prognosis in EC; however, the heterogeneous clinical and molecular landscape of this cancer makes difficult to delineate a solid and unique classification scheme to manage the EC patients adequately.^80^ Given that, the identification of novel biomarkers able to foster more precise medical approaches represents an unmet clinical requirement. In this context, miRNAs may represent an interesting opportunity as they can be obtained either by endometrial biopsy as well as blood sampling. Indeed, being miRNAs levels highly context‐specific, they could easily show a peculiar deregulation based on specific tumor characteristics. Given that, ideally, miRNAs could potentially be integrated in the current prognostic outline. In this regard, the present review aimed to describe the miRNAs reported as associated with existing international classifications and molecular features in EC. However, considering that most of reports have investigated the sole differences between tumor and normal tissue samples, the results are still limited, and their utility may currently appear scant. In addition, a wide heterogeneity in the parameters considered, as well as, in the techniques employed for miRNA analysis (as RT‐PCR, IHC, NGS) makes even more difficult to reach a general consensus among the results. Not less important is the lack of standardized protocols (including sample collection, type of biological matrix, RNA extraction and techniques) which adds challenges in comparing the results from independent studies. Taken all these aspects together, it is easy to understand why no clinical translation has yet happened, and further extensive research will be mandatory to define reliable miRNAs‐candidate biomarkers. However, we should also be aware that it would be particularly challenging to identify one or a few miRNAs able, by themselves, to accurately stratify EC patients based on molecular or clinical features. This aspect is also important when considering the analysis of the TCGA databases, which, as mentioned, is attracting remarkable interest. Indeed, it is not unusual that single databases (ie, database of mRNAs or miRNAs) are considered independently; however this is not the best approach since miRNAs strictly regulate gene expression and, thus, the analysis of single datasets by themselves may allow to miss important results.^81^ This combined approach has been applied to EC survival but the same has been poorly employed for patient stratification.^81^ These large databases, if correctly analyzed and interpreted, can represent unprecedent tools to decipher the EC heterogeneity. Nevertheless, even in this case, multiple variables (including, but not limited to, somatic mutations, IHC and clinical parameters) should also be taken in consideration. An attempt has been done by Wang et al,^64^ and by Lu et al,^82^ who identified signatures of miRNAs to predict the prognosis of ECs. When the authors combined the clinicopathological features with miRNAs, the prediction ability of the model improved. It is noteworthy to highlight that the prognostic miRNAs‐based‐model by Wang and colleagues was also associated with the TCGA classification scheme, showing that the model could also represent an alternative approach or an integration to the EC molecular classification. In the near future, these sophisticated models could ease a more precise clinical and molecular characterization, thus, promoting benefits for those ECs that are classified with intermediate prognosis and hard to adequately manage. Indeed, since no reliable prognostic markers are available, this uncertain group remains the biggest challenge, even for expert pathologists.

Another interesting aspect to consider is the liquid biopsy and the circulating miRNAs. Last decade has seen an increasing interest in noninvasive approaches to detect and classify cancer. Some cancer types, as lung and breast cancer, have been widely investigated, allowing to develop the first FDA‐approved tests based on circulating biomarkers.^83^ Other tumors, including EC, have suffered of limited research. The consequence is that within the EC panorama the research on liquid biomarkers remains in its embryonal phase and no reliable miRNA‐candidates have been identified yet. Indeed, only three studies, with very small cohorts of patients, have, so far, evaluated circulating miRNAs and prognostic features but, however, paving the way for further investigations. Interestingly, there are no studies analyzing miRNAs alterations and therapeutic response, but they definitely would be worth to better understand the biological mechanisms of clinical response in EC.

**FIGURE 2:**
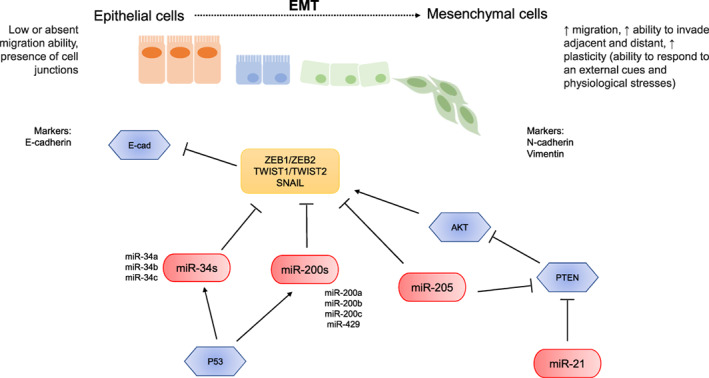
Summary of the potential targets of miRNAs with higher consensus among the different papers included in this review. Among the miRNAs, miR‐34 and miR‐200 families, miR‐205, miR‐21 have been proposed as involved in regulation of several genes involved in epithelial mesenchymal transition (EMT). The EMT is a biological process by which epithelial cells lose their cell polarity and cell‐cell adhesion, and gain migratory and invasive properties. E‐cad, E‐cadherin [Color figure can be viewed at wileyonlinelibrary.com]

Based on the data reported in our work, the most appealing miRNAs in EC belong to the miR‐200 and the miR‐34 families. So far, indeed, multiple research groups have showed the association of these miRNAs with EC characteristics. These correlations could be due to their involvement in the EMT process which is known to play an important role in EC progression, metastases and recurrence and being one of the cancer escape routes to medical treatments; Figure 2 depicts the main miRNAs involved in EMT in EC. However, additional studies are strongly warranted in order to elucidate the role and to clarify if one or more members of miR‐34 and miR‐200 families may represent reliable diagnostic, prognostic biomarkers or potential therapeutic targets.

In conclusion, besides the wide efforts done in characterizing miRNAs in EC, their utility is currently still scarce, and a global effort should be considered as mandatory to achieve a clinical translation of tissue miRNAs and, possibly, “liquid miRNAs” into the clinic.

## CONFLICT OF INTEREST

The authors declare no potential conflict of interests.

## Supporting information


**Appendix S1**: Supporting Information.
