# Supplementary material for: Can miRNAs be useful biomarkers in improving prognostic stratification in endometrial cancer patients? An update review
Source: Int J Cancer. 2021 Nov 17;150(7):1077–90. doi: 10.1002/ijc.33857 (PMC9298718; doi:10.1002/ijc.33857)
Supplement: Supplementary file 1 — Appendix S1: Supporting Information. [file IJC-150-1077-s001.pdf]

Title: Can miRNAs be useful biomarkers in improving prognostic stratification in endometrial cancer patients? An update review

Gloria Ravegnini [1+], Francesca Gorini [1], Eugenia De Crescenzo [2,3], Antonio De Leo [4,5,6], Dario De Biase [1,6], Marco Di Stanislao [2,3], Patrizia Hrelia [1], Sabrina Angelini [1] , Pierandrea De Iaco [2,3,6], Anna Myriam Perrone [2,3,6]

Table of Contents:

Supplementary table 1. Main circulating miRNAs identified by comparing endometrial cancer and healthy controls

Supplementary table 1. Main circulating miRNAs identified by comparing endometrial cancer and healthy controls			
**Author, year, reference**	**Number of samples analyzed**	**Type of body fluid**	**Main results**
Torres et al, 2013 [36]	34 EEC vs 14 HCs	Blood	miR-99a, miR-100, miR-199b: ↑ in ECs compared with HCs
Jia et al, 2013 [57]	33 EECs vs 42 HCs	Blood	miR-186, miR-204, miR-222, miR-223: ↑ in ECs compared with HCs
Gao et al, 2016 [56]	50 ECs, 50 benign lesions, 50 HCs	Blood	miR-21: ↑ in ECs compared to HCs: ↑ in ECs compared to benign lesions
Benati et al, 2017 [55]	45 ECs vs 30 HCs	Blood	miR-203: ↑ in ECs compared to HCs
Fan et al, 2021 [54]	92 ECs vs 102 HCs	Blood	miR-143-3p, miR-195-5p, miR-20b-5p, miR-204-5p, miR-423-3p, miR-484: ↑ in ECs compared with HCs
Ghazala et al, 2021 [59]	36 ECs vs 36 HCs	Blood	miR-27a, miR-150-5p: ↑ in ECs compared with HCs

Abbreviations

↑: over-expression; EC: Endometrial cancer; EEC: endometriod EC; HC: healthy control
